# Two-stage cooperative optimisation of signal timing and vehicle trajectories for eco-driving at signalised intersections under HDV/CAV/CCAV mixed traffic

**DOI:** 10.1371/journal.pone.0359104

**Published:** 2026-10-27

**Authors:** Ruoyi Wen, Wei Chu, Xiaoyun Liu, Yunxin Huang, Zexiao Chen, Huiqiang Luo

**Affiliations:** 1 Yunnan Yunling Highway Science and Technology Co., Ltd., Kunming, Yunnan, China; 2 Faculty of Transportation Engineering, Kunming University of Science and Technology Co., Ltd., Kunming, Yunnan, China; University of Cincinnati, UNITED STATES OF AMERICA

## Abstract

The transportation sector accounts for approximately 20% of global energy consumption and is responsible for nearly 25% of global energy-related CO_2_ emissions, 75% of which come from road transport. Global energy consumption and CO_2_ emissions from transportation are projected to increase by more than 50% by 2030, and this energy consumption is causing environmental damage. As autonomous vehicles become more widespread, energy consumption issues in the new context of human-machine mixed driving are gradually attracting attention. To reduce energy consumption in emerging human-machine hybrid traffic flows and promote ecological driving, while accounting for the trend toward mixed traffic comprising autonomous and manually driven vehicles, this study proposes a method to guide manually driven vehicles at signalized intersections by having autonomous vehicles lead them. An ecological driving strategy based on a mixed-traffic-following model is established. The study designed a two-way, four-lane, cross-shaped, connected intersection scenario, employing a two-stage collaborative optimisation strategy for traffic signals and vehicle trajectories to coordinate the driving states of various vehicle types within the mixed fleet, thereby optimising following distances and speeds. By constructing vehicle energy consumption models and trajectory optimization models, effective reductions in vehicle energy consumption were achieved. Simulation results indicate that as the penetration rate of autonomous vehicles increases, the number of stops and energy consumption of vehicles in mixed traffic flow significantly decrease, validating the effectiveness of the proposed strategy and providing strong support for reducing carbon emissions.

## 1. Introduction

Globally, incomplete data from around the world indicates that the transportation sector accounts for a high proportion of energy consumption, resulting in the emission of large amounts of greenhouse gases (GHGs) directly into the atmosphere and causing serious environmental problems [1]. The transportation sector accounts for approximately 20% of global energy consumption and is responsible for nearly 25% of global energy-related CO_2_ emissions, 75% of which come from road transport. Furthermore, due to population and economic growth, global energy consumption and CO_2_ emissions from transportation are projected to increase by more than 50% by 2030 [1,2]. These greenhouse gases not only contribute to climate change but also degrade land and water resources. Scholars from all walks of life have focused on how to minimise vehicle energy consumption and reduce environmental impact.

Scholars have proposed a series of technical measures, including signal control, eco-driving, and the adoption of alternative fuels, to improve the energy utilisation efficiency of vehicles [3]. Eco-driving, a key technology for reducing energy consumption and emissions, is currently benefiting from the widespread adoption of intelligent network technology, which can mitigate the effects of rapid vehicle acceleration and deceleration by providing speed guidance without altering vehicle structure, thereby reducing energy consumption and lowering pollutant emissions. Eco-driving refers to the optimal control problem (OCP) that maximises energy efficiency from a vehicle driving perspective [4]. Specifically, by accounting for surrounding traffic conditions, eco-driving enables road vehicles to achieve superior fuel economy [5]. It integrates multiple objectives—including energy efficiency, safety, and comfort—to minimise overall driving costs through systematic optimisation. In hybrid vehicles, a heterogeneous multi-agent reinforcement learning framework decomposes eco-driving into the cooperative optimisation of adaptive cruise control and energy management strategies. This approach significantly reduces fuel consumption and battery degradation costs while maintaining the following performance [6]. For connected autonomous vehicles, eco-driving avoids selfish optimisation by enabling global speed planning and real-time tracking, integrating longitudinal and lateral control to enhance energy efficiency. Compared to human driving, this approach improves fuel economy [7]. In electric vehicles, a minimal principle-based algorithm models eco-driving as an optimal control problem, accounting for diverse traffic and road constraints to achieve significant energy savings [4]. Thus, eco-driving as an independent objective effectively coordinates multi-domain requirements, enhancing overall driving economy and sustainability. With the rapid development of networked autonomous driving technology, the technology is gradually maturing, expanding the scope of eco-driving implementation. Guiding manually driven vehicles to achieve optimal trajectories using controllable self-driving vehicles is an innovative approach to addressing excessive energy consumption. To maximise the guidance effect, self-driving and manually driven vehicles need to form a mixed fleet and arrange themselves in an orderly manner according to specific rules and distances, maintain the required speed and safety distance, and improve traffic flow efficiency through joint coordination, thereby further enhancing the effect of eco-driving.

Recent studies have made significant progress in predictive control strategies for single-vehicle eco-driving. For instance, Magzhan et al. [8] proposed a look-ahead driving scheme to improve traffic flow efficiency at intersections, while Magzhan et al. [9] further developed an adaptive horizon MPC framework to balance computational cost and prediction accuracy. Although these methods effectively optimize individual vehicle trajectories based on signal phase and timing (SPaT) information, they primarily assume a homogeneous traffic environment. They often overlook the dynamic interactions and heterogeneous behaviors between connected automated vehicles (CAVs) and human-driven vehicles (HDVs), which is a critical characteristic of the transition phase in real-world urban traffic.Single-vehicle trajectory generation algorithms are a key research in eco-driving at urban signalised intersections. Barth et al. proposed a driving strategy that monitors traffic speed, density, and flow rate to provide drivers with dynamic speed recommendations 10% to 20% fuel savings and CO_2_ emission reductions without a significant increase in delays [10]. In addition, Hao et al. proposed an eco-driving system with adaptive signal timing that guides vehicles through intersections by optimising vehicle speed trajectories [11]. Zhou et al. proposed a trajectory-planning method that simultaneously optimises travel time, safety level, and fuel consumption, thereby allowing CCAVs to avoid complete stops at intersection segments [12,13]. Altan et al. proposed the Glide-Path energy-saving driving trajectory optimisation method, which divides a vehicle’s trajectory through an intersection into four phases and uses segmented trigonometric functions as the acceleration curve model to generate a smooth vehicle trajectory, thereby effectively reducing energy consumption [14]. The above studies have optimised the ecological driving trajectory of a single vehicle from various perspectives but have ignored the influence of the car ahead on the target vehicle. Moreover, the current research primarily focuses on the pure networked-vehicle environment. However, the popularisation of CAVs requires a process: the first emergence of a mixed traffic flow composed of HDVs and CAVs with different levels of vehicle intelligence, due to uncertainty in HDV driving behaviour, which results in a more complex eco-driving problem. To realise eco-driving, CAV vehicles need to guide HDVs. Currently, some scholars have proposed a hybrid fleet approach, in which the advanced information-sensing capabilities of CAVs guide HDVs to realise eco-driving across the entire fleet.

In research on fleet eco-driving strategies, Wu et al. proposed a dynamic eco-driving model that considers the vehicle ahead’s state and used a nonlinear programming algorithm to obtain the optimal speed combination and the optimal driving trajectory for the fleet [15]. The results show that a fleet of self-driving vehicles is suitable for the first strategy. In contrast, the driver-led fleet is better suited to the second strategy, and both eco-driving strategies can reduce energy consumption [16]. Wang et al. proposed a two-stage eco-driving control strategy for hybrid driving scenarios using hybrid electric vehicles as the study’s object, which reduced fuel consumption by 34.10%. In contrast, pollutant emissions (HC, CO, and NOx) were also reduced to some extent [17]. Yang et al. explored the relationship between micro-parameters and EV energy consumption under two traffic conditions: uniformly distributed traffic and minor fluctuations in the traffic system. The results showed that a vehicle-following model that accounts for the probability of traffic interruptions can reduce EV energy consumption [18]. Li proposed a speed-maximization model for autonomous driving trajectory optimization and a time-independent optimization method suitable for appointment-based intersection control, with the aim of improving vehicle throughput and the coordinated performance of intersections. [19,20] Furthermore, to address the uncertainty in traffic flow, data-driven and stochastic approaches have also been explored. Bakibillah et al. [21] presented an event-driven stochastic eco-driving strategy that learns from self-driving data to optimize velocity profiles. While such learning-based methods are effective at adapting to stochastic traffic events, they may lack the rigorous real-time coordination mechanisms required for mixed-traffic platoons. In contrast, our work focuses on a model-based collaborative optimization that explicitly considers the ‘Vehicle-Road-Signal’ coordination, ensuring both energy efficiency and system stability in a heterogeneous fleet. It is also noted that specific energy consumption models have been developed for electric vehicles (EVs) to maximize battery efficiency [22]. Although previous studies have proposed methods to address the ecological driving problem in mixed-traffic convoys at intersections, due to the complexity of the constraints and the computational demands of the algorithms, it remains to be verified in practice whether they can respond in real time to changing traffic environments.However, the current study focuses on the general longitudinal dynamics and mixed traffic flow characteristics, aiming to establish a universal cooperative optimisation framework applicable to various vehicle types, including both internal combustion engine vehicles and EVs, in a connected environment.

Despite the aforementioned advancements, a critical challenge persists during the transition to full automation: the coexistence of heterogeneous vehicle types. In mixed-traffic environments, the stochastic and often aggressive driving behaviours of Human-Driven Vehicles (HDVs) can disrupt the smooth trajectories of Connected Automated Vehicles (CAVs). This disruption not only compromises traffic stability but also negates the potential energy savings that CAVs could otherwise achieve in a homogeneous fleet. Current eco-driving strategies, often designed for either pure HDV traffic or idealised CAV platoons, fail to adequately address the negative externalities that HDVs impose on the cooperative system. Previous studies have primarily focused on isolated aspects of the problem. Some researchers optimized signal timings without considering the micro-level trajectory adjustments of individual vehicles [10,11]. Others investigated vehicle-following models but often assumed a homogeneous traffic flow or ignored the upstream traffic state constraints [15,16]. Furthermore, while fleet-based eco-driving has been explored, there is a lack of research that simultaneously considers the ‘Vehicle-Road-Signal’ coordination under varying CAV penetration rates. Specifically, there is a gap in establishing a unified framework to guide HDVs, leveraging the controllability of CAVs to form an energy-efficient mixed convoy.

To bridge this gap, this study proposes a Two-Stage Collaborative Optimization Strategy for eco-driving at signalized intersections in mixed traffic environments. The specific contributions of this work are threefold: 1. A Mixed-Traffic-Following Model: Unlike traditional models that treat all vehicles uniformly, we explicitly model the four distinct following scenarios (HDV-HDV, HDV-AV, AV-HDV, AV-AV) to capture the heterogeneity in driving behaviors. 2. Integrated Signal and Trajectory Optimization: We formulate a bi-level optimization problem. The upper level optimizes the signal timing to maximize green light utilization, while the lower level optimizes the leading vehicle’s trajectory to minimize energy consumption, ensuring coordination between infrastructure and vehicles. 3. Quantitative Analysis of Energy Impact: Using MATLAB simulations, we demonstrate how varying CAV penetration rates affect the energy consumption of the entire mixed fleet. Our results provide specific quantitative evidence that the proposed strategy can significantly reduce stops and fuel consumption, even in the presence of disruptive HDV behaviors.

Ref [23] served exclusively as the foundation for the “microscopic parameter calibration” in the mixed traffic flow environment, ensuring the physical realism of our simulation. In contrast, the current manuscript proposes a comprehensive “system-level cooperative optimization framework” aimed at solving the eco-driving problem.

1. Carried Over:

The only aspect directly inherited from Ref. [23] is the calibration of the microscopic traffic flow parameter. In Ref [23], We conducted a rigorous analysis to identify the specific behavioural parameters (such as reaction times, desired headways, and acceleration/deceleration characteristics) for HDVs, CAVs, and CCAVs in a mixed-traffic environment. In the current manuscript, we have utilized these calibrated parameters (specifically for the IDM, ACC, and CACC models mentioned in Section 2.2) solely to ensure that the simulation environment is realistic and that the vehicle dynamics are consistent with real-world observations.

2. New (The Novelty):

Signal Timing Layer (New Contribution): Ref [23] was purely focused on traffic flow stability analysis and did not involve any signal timing optimization. The current work introduces a novel bi-level (two-stage) cooperative optimization model. Specifically, we have developed an upper-level signal control optimization model (Section 2.3.2) that was entirely absent in Ref [23]. This model dynamically coordinates green-light timing and phase sequence based on the approaching platoon’s state, a fundamental extension beyond the scope of our previous stability analysis.

Mixed-Flow Car-Following Formulation (Strategic Evolution): While we use the mathematical forms of IDM and PATH models (standard in the field) with parameters calibrated in Ref [23], the cooperative driving strategy implemented in this paper is entirely new. Ref [23] analyzed how vehicles follow each other in equilibrium. In contrast, this study proposes a proactive strategy where CAVs act as “platoon leaders” to guide HDVs, and we have formulated a new objective function that couples vehicle trajectory planning with signal phase constraints (Equations 13–27). This transforms the model from a descriptive tool (Ref [23]) into a prescriptive optimization tool.

PSO Solution Algorithm (Functional Adaptation): In Ref [23], PSO was used for parameter identification (inverse problem). In this manuscript, we have completely redesigned the PSO algorithm to solve a two-stage joint optimization problem (direct problem). The algorithm now simultaneously optimizes discrete signal timing variables and continuous vehicle speed profiles. The solution space, objective function (which now includes monetary cost and energy consumption), and the convergence criteria are fundamentally different.

Evaluation Metrics (Expansion of Scope): Ref [23] evaluated traffic flow stability and equilibrium states. The current work evaluates Eco-driving performance (energy consumption reduction, specifically the 32.6% reduction discussed in Section 4.3) and dynamic delay under the cooperative optimization strategy.

## 2. Methods

This study analyses the characteristics of mixed fleets from a traffic-flow perspective and constructs an ecological driving model. In a mixed fleet, the following behaviors among different models will affect the overall operating status of the fleet, and it is necessary to portray the following behaviors within the mixed fleet accurately; to realize the ecological driving of the mixed fleet, it is essential to explore the energy consumption of each model within the fleet, and to achieve uniformity of measurement, it is necessary to consider the characteristics of each model to establish a consistent model of following of vehicles in mixed traffic flow, and to carry out an evaluation of the effect of ecological driving.

### 2.1 Human-machine mixed driving car-following behaviour

In mixed traffic flow scenarios where both autonomous vehicles and human-driven vehicles coexist on roads, car-following behaviour is classified into four types: human-driven vehicle following human-driven vehicle (HDV-HDV), human-driven vehicle following autonomous vehicle (HDV-AV), autonomous vehicle following human-driven vehicle (AV-HDV), and autonomous vehicle following autonomous vehicle (AV-AV) (Fig 1).

**Fig 1:**
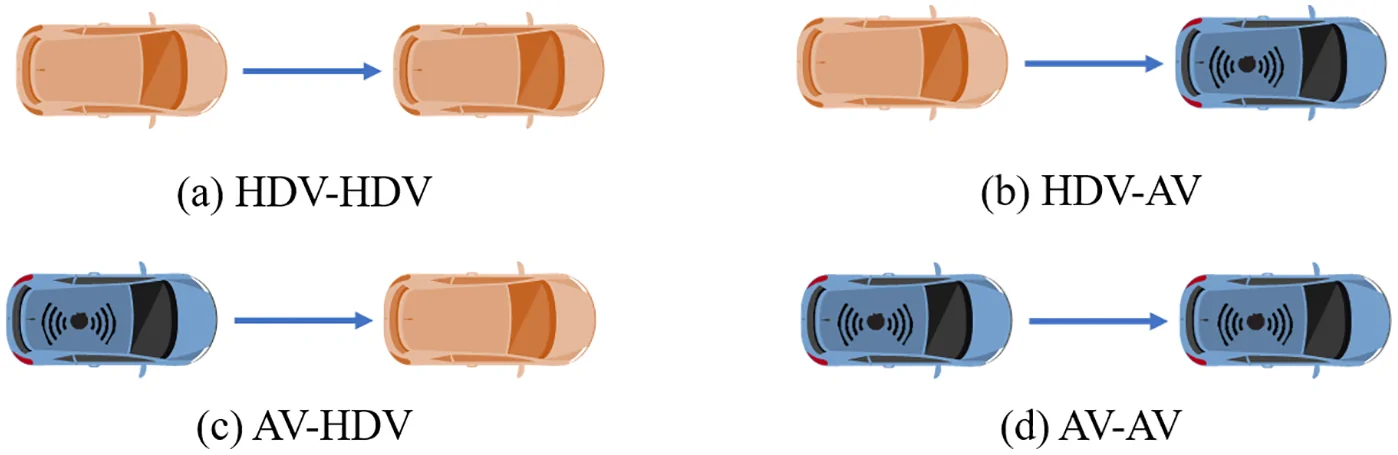
Types of vehicle following behaviour in a mixed traffic environment.

To simplify the formulation, HDV is used to refer to a human-driven vehicle, CAV to refer to a cyber-connected automated vehicle, CCAV refers to Cooperative Connected-Automated Vehicle, and CAV and CCAV are collectively referred to as autonomous vehicles (AV). Among them, CAV supports adaptive cruise control and relies on single-vehicle sensors to achieve car-following. In contrast, CCAV supports cooperative adaptive cruise control and realises multi-vehicle cooperative optimisation through V2V communication.

Among these, the IDM model is used to describe the following behaviour of human-driven vehicles (HDVs) in mixed traffic flows [23,24]. The cooperative adaptive cruise control (CCAC) and adaptive cruise control (ACC) models proposed by the PATH Laboratory describe the following behaviour of connected autonomous vehicles (CAVs) in mixed traffic flows. In CAV following mode, the ACC model describes CAV following behaviour; in CCAV following mode, the CACC model describes CCAV following behaviour [25]. (Fig 2).

**Fig 2:**
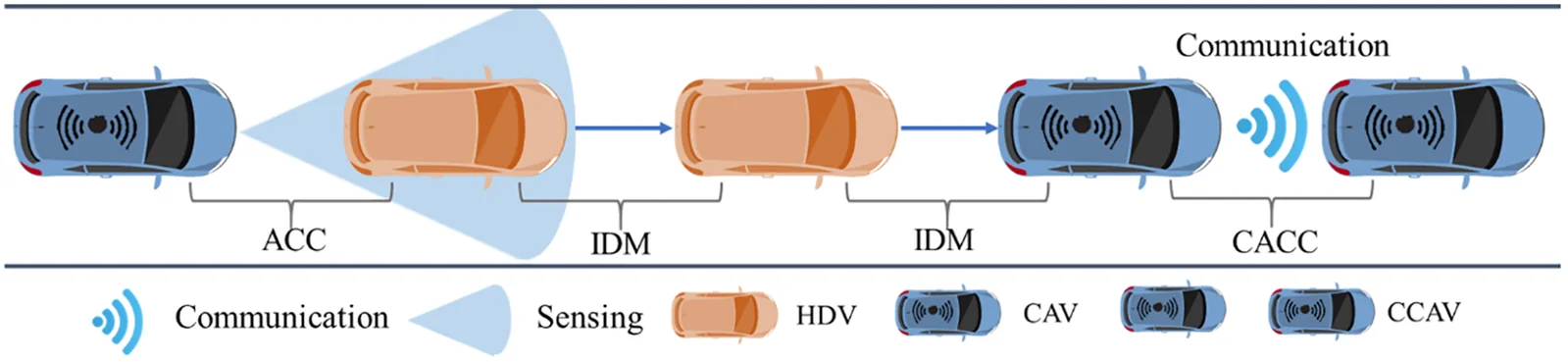
Schematic illustration of vehicle following behaviour in mixed traffic environment.

### 2.2 Mixed‑traffic‑following model

The IDM model captures the vehicle’s following behaviour to obtain the following acceleration and, based on this, determines the acceleration, velocity, and displacement parameters for the next time step, thereby enabling microscopic modelling of macroscopic traffic flow [24]. The IDM model is as follows:


$$\begin{array}{c} a_{n}\left(t\right)=a_{max}\left[1-{\left(\frac{v_{n}\left(t\right)}{{\tilde{v}}_{n}}\right)}^{\delta }-{\left(\frac{{\tilde{l}}_{n}\left(t\right)}{l_{n}\left(t\right)}\right)}^{\text{2}}\right], \end{array}$$ (1)

$$\begin{array}{c} {\tilde{l}}_{n}\left(t\right)=l_{\text{0}}+v_{n}\left(t\right)\tilde{T}+\frac{v_{n}\left(t\right)\Delta v\left(t\right)}{2\sqrt{a_{max}b}}, \end{array}$$ (2)

$$\begin{array}{c} \Delta v\left(t\right)=v_{n-1}\left(t\right)-v_{n}\left(t\right), \end{array}$$ (3)

In the above equation: δ is the free stream acceleration index; $a_{n}\left(t\right)$ is the output acceleration of the model at time t; $a_{max}$ is the maximum acceleration; $v_{n}\left(t\right)$ Is the instantaneous speed of the vehicle at time t; ${\tilde{v}}_{n}$ is the desired speed; ${\tilde{l}}_{n}\left(t\right)$ is the desired headspace; $l_{n}\left(t\right)$ Is the distance between the front and rear of the two cars at the moment t; $v_{n-1}\left(t\right)$ is the speed of the front car at time t; $l_{\text{0}}$ is the stopping distance between the two vehicles; $\tilde{T}$ is the desired head time distance; b is a comfortable deceleration value; Δv(t) is the difference in speed between the two cars before and after the time t.

Here are the parameters of the IDM model (Table 1) [26].

**Table 1:** IDM model parameters.

Parameter	Implications	Unit	Values
δ	free-flow acceleration index	–	4
$\tilde{T}$	desired headway	s	1.5
${\tilde{v}}_{n}$	expected speed	$m\cdot s^{-1}$	25
$a_{\text{max}}$	maximum acceleration	$m\cdot s^{-2}$	1.4
b	comfort deceleration	$m\cdot s^{-2}$	2
$l_{\text{0}}$	parking space	m	2

The adaptive cruise control (ACC) model is constructed as follows [15]:


$$\begin{array}{c} \overset{―}{v}=v_{i}-v_{r}, \end{array}$$ (4)

$$\begin{array}{c} a=a_{c}, \end{array}$$ (5)

$$\begin{array}{c} a_{c}=bound\left(-0.4\cdot \overset{―}{v},2,-2\right), \end{array}$$ (6)

In the above equation $v_{i}$, is the vehicle speed; $v_{r}$ is the road speed limit; $\overset{―}{v}$ is the speed error; a is the vehicle acceleration; $a_{c}$ is the vehicle acceleration in speed control mode. The +2 and −2 in $a_{c}=bound\left(-0.4\cdot \overset{―}{v},2,-2\right)$ represent the vehicle’s maximum and minimum accelerations, respectively, as shown in Formula $a_{c}=bound\left(-0.4\cdot \overset{―}{v},a_{max},a_{min}\right)$.

The cooperative adaptive cruise control CACC model is constructed as follows [15]:


$$\begin{array}{c} \overset{―}{v}=v_{i}-v_{r}, \end{array}$$ (7)

$$\begin{array}{c} a_{c}=bound\left(-0.4\cdot \overset{―}{v},2,-2\right), \end{array}$$ (8)

$$\begin{array}{c} l_{d}=T_{b}\cdot v_{i}, \end{array}$$ (9)

$$\begin{array}{c} l_{e}=l_{d}-l_{r}, \end{array}$$ (10)

$$\begin{array}{c} e=x\left(i-1\right)-x\left(i\right)-Tv_{i}, \end{array}$$ (11)

$$\begin{array}{c} a_{i}=k_{\text{0}}a_{i-1}+k_{\text{1}}e+k_{\text{2}}\left(v_{i-1}-v_{i}\right), \end{array}$$ (12)

In the above equation: $l_{r}$ is the desired headway of the rear vehicle, that is, the minimum safe distance between vehicles when stationary (the distance between bumpers when parked).; $l_{e}$ is the headway error between the rear vehicle and the front vehicle; $l_{d}$ is the distance between the front of the rear vehicle and the front of the vehicle in front, dynamic spacing that takes speed into account.; $T_{b}$ is the desired time interval of the rear vehicle; T is the time interval between the front vehicle and the rear vehicle; and e is the inter-vehicle distance error; x(i) is the position of the vehicle i; $a_{i}$ is the acceleration of the vehicle i; $k_{\text{0}}$ is the acceleration weight; $k_{\text{1}}$ is the workshop distance error weight; $k_{\text{2}}$ Is the velocity difference weight? The model parameters calibrated by foreign scholars have been verified by real-vehicle experiments [27], in which $k_{\text{0}}=1.1$$k_{\text{1}}=0.23$$k_{\text{2}}=0.07$, can more objectively reflect the effect of CCAV on the dynamic characteristics of mixed traffic flow [28].

### 2.3 Co-optimisation of traffic signals and vehicle trajectories in two phases

#### 2.3.1 Co-optimisation strategy.

Most previous studies have optimised CAV trajectories to minimise the autonomous vehicle’s driving performance. Vehicles approaching a red light need to stop and wait, then accelerate through the intersection when the light turns green. However, this process reduces the vehicle’s travel time between two intersections. However, it increases vehicle queuing time, and as the number of intersections increases, the cumulative increase in vehicle waiting time is greater. The total travel time is longer of due toincreased vehicle waiting time, which in turn reduces the intersection’s total capacity. Using the two-phase cooperative optimisation control strategy proposed in this study, through trajectory optimisation, the CAV can decelerate or accelerate in advance to avoid stopping and queuing, which significantly reduces the vehicle waiting time, and through the optimisation of the signal timing scheme, it can effectively reduce the vehicle delay and improve the total intersection capacity (Fig 3).

**Fig 3:**
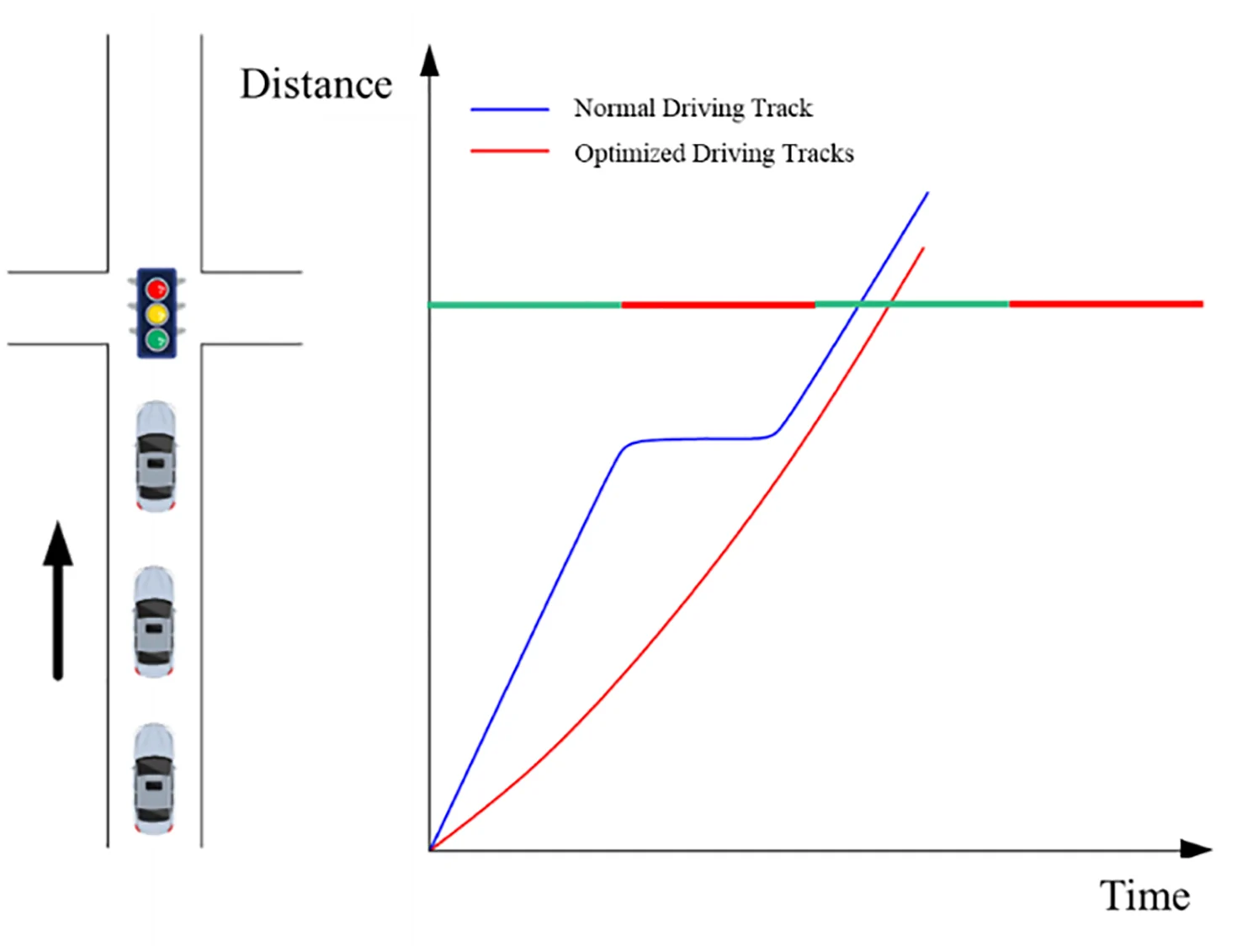
Schematic diagram of vehicle trajectory comparison.

The two-stage joint optimisation strategy aims to enhance the utilisation efficiency of green time while minimising vehicle delays and smoothing vehicle trajectories to reduce energy consumption. This is a comparison of the green light time utilisation rate between the actual-state signal control and the optimisation strategy proposed in this study (Fig 4).

**Fig 4:**
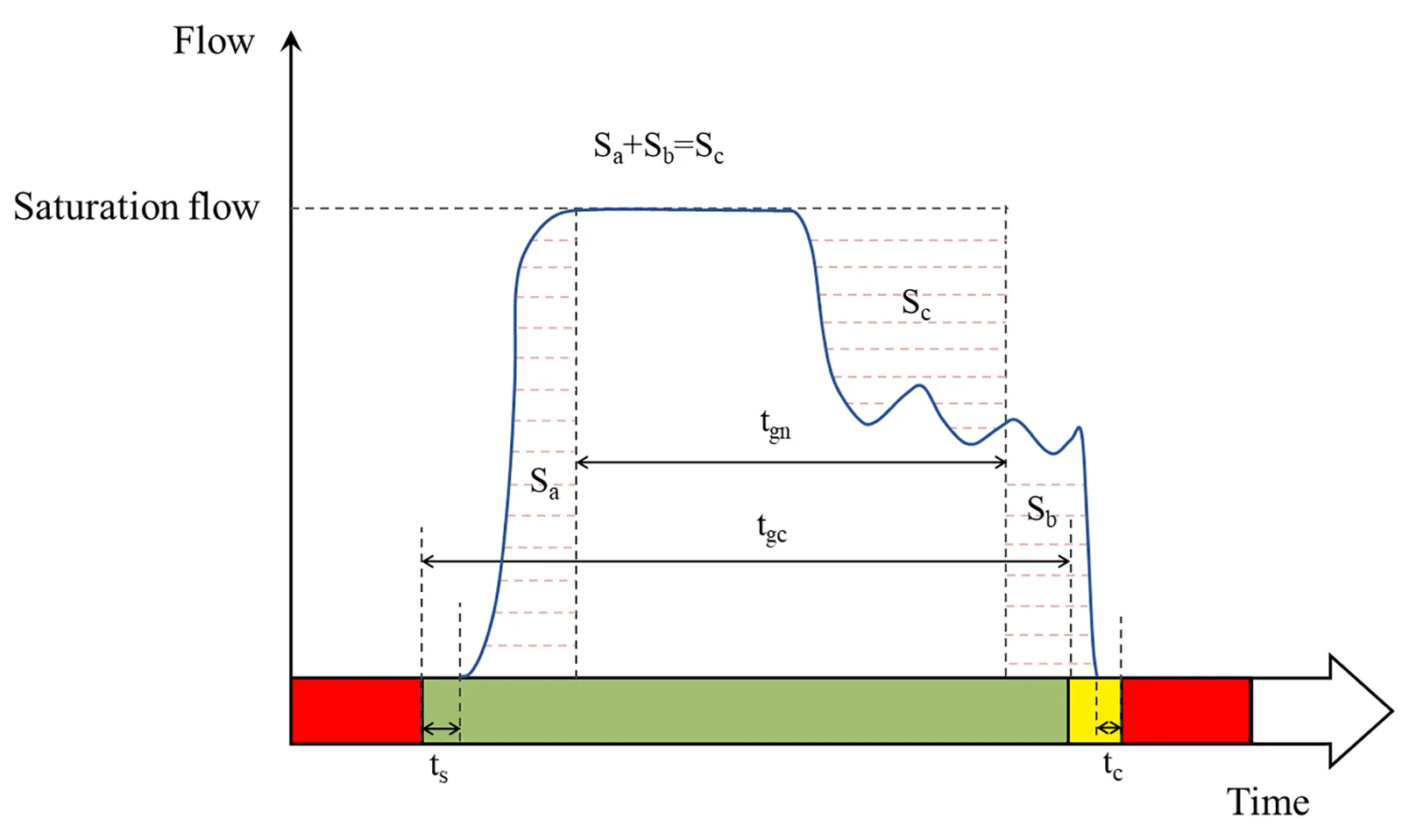
Schematic comparison of green light time utilisation rate.

In Fig 4, the blue curve shows the vehicle release rate at the intersection under the current signalling control strategy, i.e., the number of vehicles released from the queue state per unit of time. In the initial stage, when the vehicle release rate reaches the saturation flow rate and all queued vehicles have passed through the current signalised intersection, the vehicle release rate gradually decreases to the arrival rate, which is usually lower than the saturation flow rate. The black-dashed line shows the vehicle release rate at the intersection under the optimisation strategy proposed in this study. In the proposed approach, the lead vehicle’s trajectory is controlled so that it arrives at the intersection at a specified speed at the initial moment of the green light. Since the departure time of the convoy’s lead vehicle is determined by the trajectory control algorithm, and the other vehicles can follow it, the departure times of all following vehicles can be estimated using the saturation flow rate. The area under the curve is the vehicle throughput during the green light period, when $S_{c}$ is equal to the sum of. Thenthis optimization strategy significantly improves the utilization of the green light time, enabling more vehicles to pass through the intersection in a shorter green light time, which effectively improves the overall efficiency of the intersection.

#### 2.3.2 Upper layer signal control optimisation model.

In the upper-level optimisation model, to minimise the intersection access delay and energy consumption cost, a value coefficient is introduced to quantify the time and energy costs into a monetary cost. The optimisation objective function can be expressed as:


$$\begin{array}{c} {min}_{t_{ij}^{f},t_{ij}^{\text{arrival}}}Cost=\eta_{\text{1}}\sum_{i}\sum_{j}(t_{ij}^{\text{arrival}}-t_{ij}^{f})+\eta_{\text{2}}\sum_{i}\sum_{j_{c}}FC\left(v_{ij_{c}}^{\text{0}},t_{ij_{c}}^{\text{arrival}},d_{ij_{c}}\right), \end{array}$$ (13)

$$\begin{array}{c} FC\left(v_{ij_{c}}^{\text{0}},t_{ij_{c}}^{\text{arrival}},d_{ij_{c}}\right)=\frac{\text{1}}{\eta_{eff}}\left[(\frac{\text{1}}{\text{2}}C_{d}A\rho {\overset{―}{v}}^{\text{2}}+\mu mgcdotd_{ij_{c}}+\frac{\text{1}}{\text{2}}m(v_{final}^{\text{2}}-(v_{ij_{c}}^{\text{0}}{)}^{\text{2}})\right] \end{array}$$ (14)

$$\begin{array}{c} \overset{―}{v}=\frac{d_{ij_{c}}}{t_{ij_{c}}^{\text{arrival}}} \end{array}$$ (15)

In the above equation $\eta_{\text{1}}$, is the coefficient of the monetary value of vehicle travel time; $\eta_{\text{2}}$ is the coefficient of monetary value of vehicle energy consumption; $v_{ij_{c}}^{\text{0}}$ is the vehicle’s initial velocity; $\overset{―}{v}$ is the average speed of the vehicle; $t_{ij}^{\text{arrival}}$ is the desired vehicle arrival time; $t_{ij}^{f}$ is the minimum time for the vehicle to arrive; $d_{ij_{c}}$ and is the planning distance travelled; $C_{d}$, A, ρ represent the vehicle’s drag coefficient, frontal area, and air density, respectively; μ, m, g represent the vehicle’s rolling resistance coefficient, vehicle mass, and acceleration due to gravity, respectively; $v_{final}$ is the target speed of the vehicle as it approaches the intersection; $\eta_{eff}$ is the overall efficiency coefficient of the vehicle’s powertrain.

The minimum total cost Cost includes the vehicle passage-delay cost $\eta_{\text{1}}\sum_{i}\sum_{j}(t_{ij}^{\text{arrival}}-t_{ij}^{f})$ and the vehicle energy-consumption cost $\eta_{\text{2}}\sum_{i}\sum_{j_{c}}FC\left(v_{ij_{c}}^{\text{0}},t_{ij_{c}}^{\text{arrival}},d_{ij_{c}}\right)$. Vehicle delay cost measures the loss of all vehicles due to delays at an intersection, and self-driving vehicle energy consumption cost reflects the energy consumed by a grid-connected self-driving vehicle as it passes through an intersection. $FC\left(v_{ij_{c}}^{\text{0}},t_{ij_{c}}^{\text{arrival}},d_{ij_{c}}\right)$ The energy consumption function of the self-driving vehicle depends on the vehicle’s initial speed and the planned arrival time.

To ensure that, in each phase, vehicles have enough time to safely pass through the intersection and avoid the green light time being too short to allow smooth passage, the green light time constraint is set. The mathematical expression is as follows:


$$\begin{array}{c} g_{min}\leq g_{p}^{k}\leq g_{max}, \end{array}$$ (16)

In the above equation $g_{p}^{k}$, is the green time duration of the p th phase in the k th signalling cycle; $g_{min}$ is the minimum green light duration; $g_{max}$ is the maximum green light duration.

To ensure that all vehicles within the intersection safely pass through the set time interval, the signal cycle constraints are set to keep the traffic signals operating cyclically with a fixed cycle, ensuring predictable traffic operations. The mathematical expression is as follows:


$$\begin{array}{c} \sum_{p}(g_{p}^{k}+r)=C, \end{array}$$ (17)

$$\begin{array}{c} s_{p}^{k}=(k-1cdotC+\sum_{i=1}^{p-1}(g_{\text{p}}^{k}+r), \end{array}$$ (18)

In the above equation r, is the clearing time; C is the signal cycle length; $s_{p}^{k}$ is the end time of the p th phase in the k th signal cycle.

Vehicle planning to arrive at the stop line must be within the phase’s green light time, in line with traffic rules and actual traffic logic, to set the vehicle arrival time constraints. The mathematical expression is as follows:


$$\begin{array}{c} s_{p}^{k}-(g_{p}^{k}+rlet_{ij}^{\text{arrival}}\leq s_{p}^{k}-r, \end{array}$$ (19)

In the above equation $t_{ij}^{\text{arrival}}$, is the planned arrival time of the j th vehicle in the i Direction.

To ensure that adjacent vehicles maintain a safe distance, prevent tailgating accidents, and maintain the safety and stability of traffic flow, the following constraint is imposed on adjacent vehicles. The mathematical expression is as follows:


$$\begin{array}{c} t_{i,j+1}^{\text{arrival}}-t_{ij}^{\text{arrival}}\geq h_{ij}, \end{array}$$ (20)

In the above equation $t_{i,j+1}^{\text{arrival}}$, is the planned arrival time of the j+1 th vehicle in the i direction; $h_{ij}$ is the headway of the j th vehicle in the i direction.

In the actual traffic, the vehicle arrival time is necessarily greater than or equal to its theoretical minimum arrival time. To comply with the basic logic of traffic operations, set constraints on the relationships among vehicle arrival times. The mathematical expression is shown below:


$$\begin{array}{c} t_{ij}^{\text{arrival}}\geq t_{ij}^{f}, \end{array}$$ (21)

#### 2.3.3 Lower-level energy consumption-based vehicle trajectory optimisation model.

The lower optimisation model is based on the optimal signal timing scheme obtained from the upper model. Taking into account the signal phase constraints, vehicle safety constraints, and the combined vehicle dynamics and energy consumption characteristics, the optimal control model is constructed to minimise the pilot vehicle’s energy consumption. Given that the motion state of the following vehicles is affected by the pilot vehicle and, under the premise of minimising the pilot vehicle’s motion, optimising the pilot vehicle can significantly improve the energy efficiency of the whole fleet. Therefore, the vehicle trajectory optimisation problem is transformed into a single-objective optimisation problem to reduce the pilot vehicle’s energy consumption. Based on this, the optimisation objective function can be expressed as:


$$\begin{array}{c} min\int_{\text{0}}^{t_{i1}^{\text{arrival}}}FC\left(t\right)dt, \end{array}$$ (22)

In the above equation FC(t), is a function of time t and represents the energy consumption rate of the pilot vehicle at the moment t, i.e., the amount of energy consumed per unit of time. By integrating FC(t) over the time interval $\left[0,t_{i1}^{\text{arrival}}\right]$, The total energy consumption of the pilot vehicle from departure to arrival at the specified position is obtained $\int_{\text{0}}^{t_{i1}^{\text{arrival}}}FC\left(t\right)dt$. By optimising the relevant variables, the pilot vehicle’s total energy consumption is minimised during this time. The modeling approach of the CMEM (Comprehensive Modal Emissions Model) was introduced as a supplement, Function $fc\left(v_{i}\left(t\right),u_{i}\left(t\right)\right)$ represents the vehicle’s instantaneous fuel consumption rate at time t [29,30]. According to Akçelik’s theory, energy consumption during steady-state cruising is primarily determined by rolling resistance and aerodynamic drag, which are represented in the formula by the polynomial term $\alpha_{\text{1}}+\alpha_{\text{2}}v+\cdot \cdot \cdot \cdot \cdot \alpha_{\text{4}}v^{\text{3}}$ with respect to velocity v. By combining Akçelik’s element-decomposition approach with CMEM’s power-demand logic, this model retains the computational simplicity of macro-statistical models while offering the high accuracy and interpretability of micro-physical models. This dual approach enables the study to accurately capture the subtle effects of frequent vehicle acceleration and deceleration on energy consumption within mixed CAV-HDV traffic flows, thereby providing a solid theoretical foundation for evaluating eco-driving strategies.

Satisfy the following constraints:


$$\begin{array}{c} {𝐱}_{i}\left(t\right)=[\begin{array}{c} s_{i}\left(t\right)\\ v_{i}\left(t\right) \end{array}]\;(t_{i}^{\text{0}}\leq t\leq t_{i}^{n}), \end{array}$$ (23)

$$\begin{array}{c} u_{i}\left(t\right)={\dot{v}}_{i}\left(t\right)\in \left[u_{min},u_{max}\right], \end{array}$$ (24)

$$\begin{array}{c} {𝐱}_{i}\left(t_{i}^{\text{0}}\right)=\left[\begin{array}{c} s_{i}\left(t_{i}^{\text{0}}\right)\\ v_{i}\left(t_{i}^{\text{0}}\right) \end{array}\right], \end{array}$$ (25)

$$\begin{array}{c} v_{min}\leq v_{i}\left(t\right)\leq v_{max}, \end{array}$$ (26)

$$\begin{array}{c} a_{min}\leq {\dot{v}}_{i}\left(t\right)\leq a_{max}, \end{array}$$ (27)

$$\begin{array}{c} RC\left({𝐱}_{i}\left(t\right),{𝐮}_{i}\left(t\right),t\right)=\zeta_{\text{1}}\cdot fc\left(v_{i}\left(t\right),u_{i}\left(t\right)\right)+\zeta_{\text{2}}\cdot u_{i}(t{)}^{\text{2}}, \end{array}$$ (28)

$$\begin{array}{c} fc\left(v_{i}\left(t\right),u_{i}\left(t\right)\right)=\left\{ \begin{array}{c} \begin{array}{ccc} \alpha_{\text{1}}+\alpha_{\text{2}}v_{i}\left(t\right)+\alpha_{\text{3}}{v_{i}}^{\text{2}}\left(t\right)+\alpha_{\text{4}}{v_{i}}^{\text{3}}\left(t\right)+\beta u_{i}\left(t\right)v_{i}\left(t\right) & if & u_{i}\left(t\right)\geq 0 \end{array}\\ \begin{array}{ccc} \alpha_{\text{1}}+\alpha_{\text{2}}v_{i}\left(t\right)+\alpha_{\text{3}}{v_{i}}^{\text{2}}\left(t\right)+\alpha_{\text{4}}{v_{i}}^{\text{3}}\left(t\right) & if & u_{i}\left(t\right)<0 \end{array} \end{array}\right. \end{array}$$ (29)

$$\begin{array}{c} \varphi \left({𝐱}_{i}\left(t_{i}^{n}\right)\right)=\zeta_{\text{3}}\cdot {\left(x_{i}\left(t_{i}^{n}\right)-d_{i}\right)}^{\text{2}}+\zeta_{\text{4}}\cdot {\left(v_{i}\left(t_{i}^{n}\right)-v_{max}\right)}^{\text{2}}, \end{array}$$ (30)

In the above equation: ${𝐱}_{i}\left(t\right)$ is the state variable of the vehicle i at moment t; $s_{i}\left(t\right)$ is the longitudinal displacement of the vehicle i at moment t; $v_{i}\left(t\right)$ is the velocity of the vehicle i at moment t; ${\dot{v}}_{i}\left(t\right)$ is the acceleration of the vehicle i at moment t; $u_{i}\left(t\right)$ is the control variable for the vehicle i at moment t; $\alpha_{\text{1}}{,\alpha }_{\text{2}},\alpha_{\text{3}},\alpha_{\text{4}}$ represents the coefficient related to vehicle resistance (rolling resistance, aerodynamic drag, and idling power consumption); β represents a coefficient related to engine efficiency; In particular, the constant term $\alpha_{\text{1}}$ represents the constant energy consumption required for the vehicle to overcome internal friction; the terms $\alpha_{\text{2}}$ and $\alpha_{\text{3}}$ correspond to the contributions of speed and the square of speed to energy consumption, respectively; and the term $\alpha_{\text{4}}v^{\text{3}}$ is specifically used to capture the physical characteristic that air resistance increases with the cube of speed. When the vehicle accelerates $u_{i}\left(t\right)\geq 0$, in addition to overcoming basic resistance, the engine must also deliver additional power to increase the vehicle’s kinetic energy; this is represented in the model by the traction power term $\beta u_{i}\left(t\right)v_{i}\left(t\right)$. ${𝐱}_{i}\left(t_{i}^{\text{0}}\right)$ is the state of the vehicle i at the initial moment $t_{i}^{\text{0}}$ and is a pre-acquired constant; $t_{i}^{\text{0}}$ is the initial time that the vehicle i enters the intersection area; $t_{i}^{n}$ is the time when the vehicle i passes through the intersection. In particular, the control dynamics parameters $\alpha_{1\;4}$ and β, as well as the multi-objective weights $\zeta_{1\;4}$, were calibrated based on the stability criterion proposed by Cai et al. and real-world vehicle driving data to ensure that vehicle behavior under mixed human-vehicle traffic conditions conforms to physical reality [31,32]. The specific weights are shown in Table 2 below.

**Table 2:** Parameter settings for the proposed optimization framework.

Symbol	$\alpha_{1}$	$\alpha_{2}$	$\alpha_{3}$	$\alpha_{4}$	β	$\zeta_{1}$	$\zeta_{2}$	$\zeta_{3}$	$\zeta_{4}$
**Value**	0.8	1.2	0.5	0.3	2.0 $s^{-1}$	0.4	0.2	0.2	0.2

### 2.4 Particle Swarm Optimisation solver algorithm

In existing research on eco-driving, the mechanisms for handling traffic uncertainties are key to determining the robustness of control strategies. Recent studies, such as Zhang et al. [33] in the context of wireless charging at signalized intersections, have primarily focused on static optimization of speed trajectories using vehicle-to-infrastructure (V2I) information. While these efforts have yielded results in energy management, they remain inadequate in addressing dynamic disturbances from mixed traffic flows (where CAVs and HDVs coexist). Meanwhile, Lu et al. [34] proposed a model predictive control (MPC)-based bimodal control strategy that effectively addresses uncertainties such as queuing and leading-vehicle interference through a “upper-level planning–lower-level feedback” architecture, demonstrating the advantages of MPC in real-time feedback control. However, MPC methods typically rely on precise predictive models and involve high computational complexity. In contrast, the two-stage Particle Swarm Optimization (PSO) framework proposed in this study offers a different solution paradigm, it distinguishes itself from traditional MPC feedback control and single-stage static optimization methods in handling the complex nonlinear characteristics of CAV-HDV interactions.

In particle swarm optimisation, each particle represents a potential solution within the solution space, characterised by two properties: position and velocity [35]. Particles adjust their positions by tracking two key points: the best position they have personally experienced (individual best, pbest) and the best position the swarm has collectively experienced (global best, gbest). Based on these two historical optimal positions, particles update their velocity and position, gradually converging toward the optimal solution (Fig 5).

**Fig 5:**
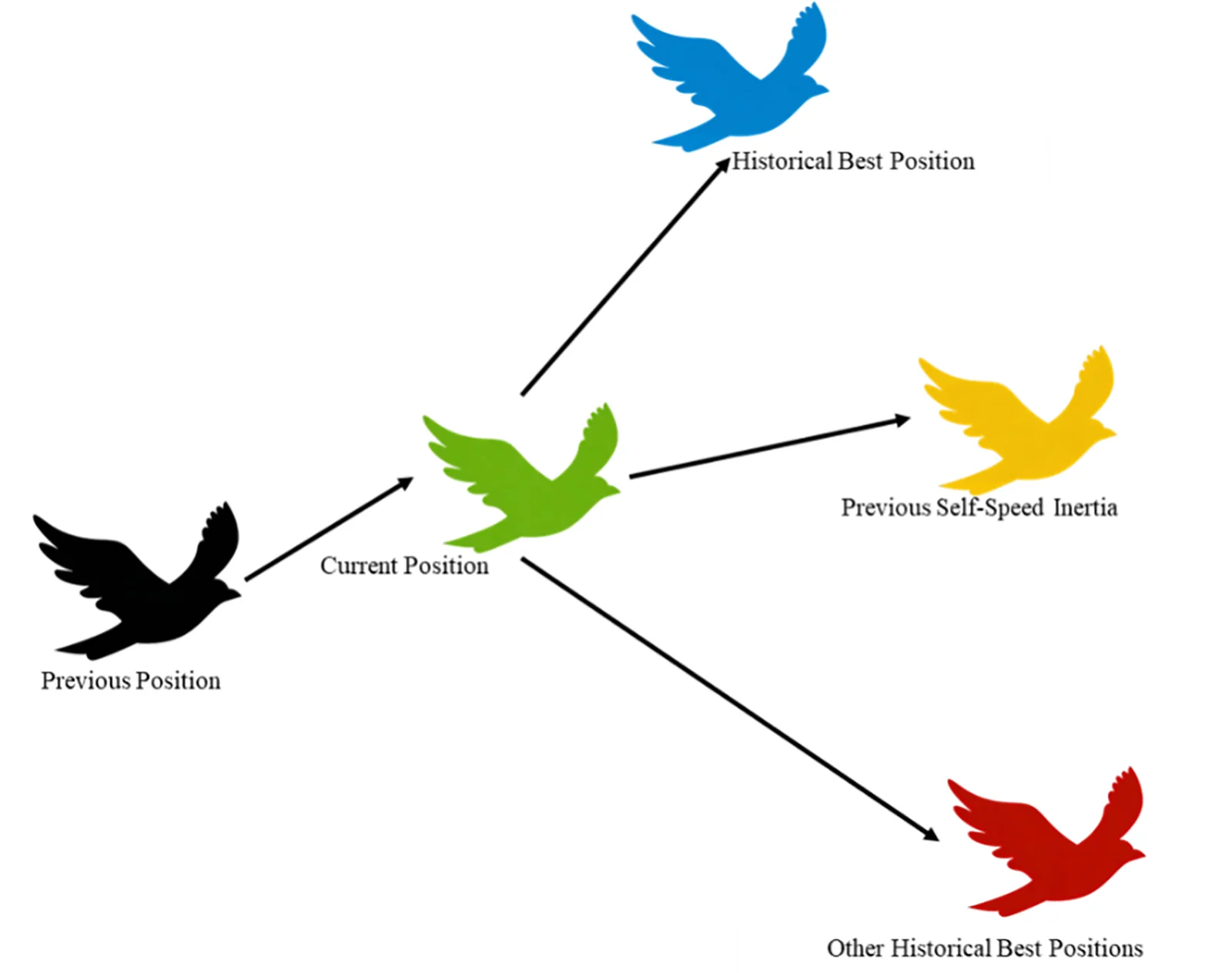
Schematic diagram of particle swarm optimisation algorithm.

When employing particle swarm optimisation to solve optimisation problems, a criterion must be established to evaluate the quality of each potential solution represented by a particle. This criterion is the fitness function, which corresponds to the objective function in optimisation problems. In vehicle trajectory optimisation, average energy consumption is commonly used as the fitness function. By adopting average energy consumption as the metric, the algorithm can search for a direction that minimizes vehicle energy consumption. Simultaneously, the algorithm determines convergence based on the fitness function values. After multiple iterations, when the particles’ fitness values no longer show significant change or exhibit only minimal variation, the algorithm is considered to have reached a converged state. At this point, a relatively optimal solution has been found. The algorithm update rules are as follows:


$$\begin{array}{c} V\text{(}t+1)=\varphi \cdot V(t)+c_{\text{1}}\cdot r_{\text{1}}\cdot \left[(P_{pbest}-X(t)\right]+c_{\text{2}}\cdot r_{\text{2}}\cdot \left[P_{gbest}-X\left(t\right)\right], \end{array}$$ (31)

$$\begin{array}{c} X(t+1)=X\left(t\right)+V(t+1), \end{array}$$ (32)

$$\begin{array}{c} \varphi =\frac{\left(\varphi_{\text{start}}-\varphi_{end})(K-k\right)}{K}+\varphi_{end}, \end{array}$$ (33)

In the equation, V(t+1) represents the particle’s velocity at time t+1; V(t) represents the particle’s velocity at time t+1; X(t) represents the particle’s position at time t+1; X(t) represents the particle’s position at time t+1; φ represents the inertia factor; $c_{\text{1}}$, $c_{\text{2}}$ represents the learning factor; is the acceleration constant; $r_{\text{1}}$, $r_{\text{2}}$ represents a random number in the interval [0,1]; $P_{pbest}$ denotes the particle’s current optimal position; $P_{gbest}$ denotes the current optimal position within the particle’s neighborhood; $\varphi_{\text{start}}$ denotes the initial inertia weight; $\varphi_{end}$ denotes the inertia weight when the maximum iteration count has been reached; k denotes the current iteration count; K denotes the maximum iteration count.

Formula 31 updates a particle’s velocity by comprehensively considering its current speed, its own historical optimal position, and the global optimal position, thereby guiding the particle to search for better solutions within the solution space. The inertia factor φ reflects how much a particle maintains its previous velocity. A larger inertia factor facilitates extensive global exploration across the search space, enabling particles to traverse different regions rapidly. A smaller inertia factor emphasises local search, allowing particles to fine-tune near current suboptimal solutions. $c_{\text{1}}$ Controls the extent to which particles learn from their historical best positions, reflecting their utilisation of experience and cognitive ability. $c_{\text{2}}$ Governs the degree to which particles learn from the global optimum, symbolising their capacity for social learning through communication and collaboration within the swarm. Random numbers $r_{\text{1}}$$r_{\text{2}}$ are introduced to enhance the algorithm’s randomness and diversity, preventing it from getting stuck in local optima.

Formula 32 updates particle positions. By continuously adjusting velocity and location, particles persistently traverse and explore the solution space, gradually converging toward the optimal solution.

Formula 33 dynamically adjusts the inertia weight. As the iteration count k increases, K It gradually decreases from an initial significant value to a smaller value. This design prioritises global search in the early stages and local search in the later stages, enhancing search efficiency and precision.

Based on existing research findings and the requirements of this paper, the parameter values for the particle swarm optimization algorithm were determined [36,37], as follows: The population size was set to 30; if the population size is too small, the algorithm is prone to getting stuck at a local optimum; if the size is too large, it will significantly increase computational complexity. A dynamic adjustment strategy is adopted for the inertia weight: the initial inertia weight $\varphi_{\text{start}}$ is set to 0.9, and the inertia weight $\varphi_{end}$, reached upon the maximum number of iterations, is set to 0.4. This configuration is designed to allow the algorithm to utilize a larger inertia weight during the early stages of iteration, guiding the particles to perform a relatively extensive global search in the solution space; in the later stages, a smaller inertia weight helps the particles make fine adjustments in local regions, thereby more accurately approaching the optimal solution. The acceleration constants $c_{\text{1}}$ and $c_{\text{2}}$ are both set to 2.0. This setting follows general best practices for particle swarm optimization and aims to balance the particles’ ability to learn from their own historical optimal positions and the global optimal position, ensuring that particles can draw on both their own experience and the swarm’s optimal information during the search process. The maximum number of iterations, K, is set to 500. This value is chosen to ensure the algorithm has sufficient iterations to converge to a favorable solution, while avoiding unnecessary waste of computational resources and time due to excessive iterations. The convergence threshold is set to 0.1%. When the rate of change in the global optimal fitness falls below this threshold, the algorithm is deemed to have converged, and the iteration process is terminated. This setting helps control computational costs and prevents the algorithm from performing excessive and ineffective computations.

## 3. Simulation experiment

### 3.1 Simulated scenario

In this study, a two-way, four-lane, typical cross-type grid intersection scenario is constructed (Fig 6). In this scenario, the intersection has eight inlets and eight outlets. The right-turn and straight traffic flows are in the same lane, and the right-turn traffic does not conflict with the rest of the traffic, so they are merged into a single phase. $\varphi_{\text{1}}$ To $\varphi_{\text{8}}$ correspond to one direction of traffic flow, respectively.

**Fig 6:**
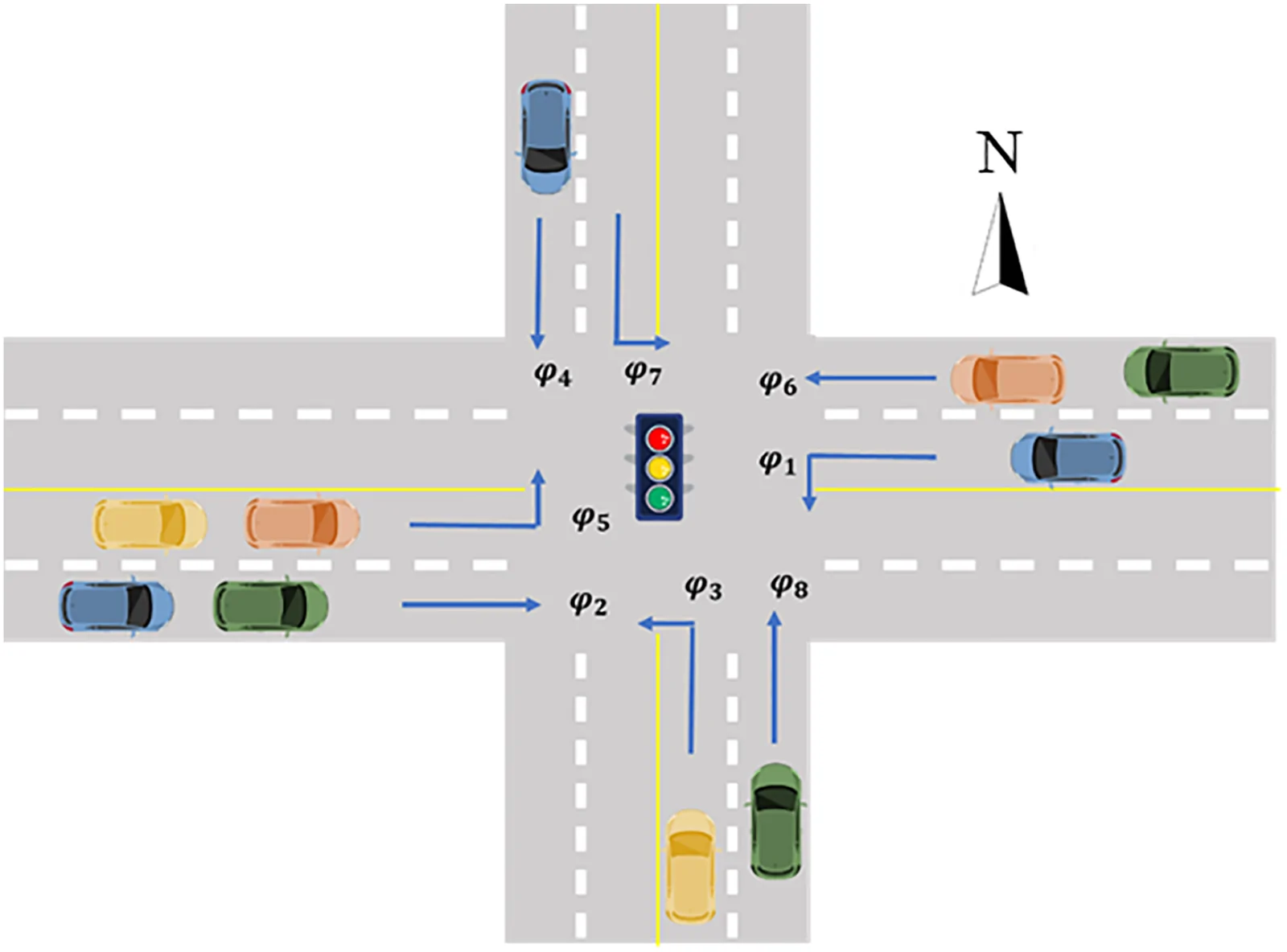
Schematic diagram of a two-way four-lane intersection.

This study makes the following assumptions about the scenario construction of the model:

(1) CCAV can obtain the operation status of HDV in real time, and then establish the collaboration mechanism between CCAV and HDV.
(2) All following vehicles follow specific following rules, and following vehicles with enormous headways will try to catch up with their leading vehicles while meeting safety restrictions. Lane-changing and overtaking behaviours are prohibited.
(3) To improve throughput efficiency and reduce vehicle delays, all CCAVs pass through intersections at desired speeds. Traffic flow oversaturation is not considered, i.e., vehicles do not experience secondary or repeated queuing, thus avoiding complex nonlinear effects caused by multiple queues;
(4) In the CCAV environment, a communication network is constructed relying on the roadside unit and the vehicle terminal for data exchange between the vehicle and the infrastructure to realise the omnidirectional transmission of traffic signal timing data. Through the infrastructure cooperative sensing mechanism, CCAV can accurately obtain the phase timing scheme and optimise the vehicle arrival time.
(5) Vehicle dynamics parameters are set as a homogenised model, i.e., all vehicles are homogeneous with the exact geometric dimensions and vehicle dynamic characteristics. All vehicles are light, with no time delay during acceleration or deceleration.
(6) The model constructed in this study will focus on single-degree-of-freedom longitudinal dynamics characteristics, i.e., only vehicle velocity and longitudinal displacement are considered. The road plane is assumed to be an ideal two-dimensional flow shape, and the effect of the longitudinal slope angle on potential energy conversion is ignored.

### 3.2 Simulation experiment settings

Based on the mixed traffic flow scenario, a two-phase joint optimisation strategy for traffic signals and vehicle trajectories is proposed to explore the effect of intelligent networked vehicles on improving access efficiency. To verify the model’s effectiveness, mixed traffic flow simulation experiments are conducted using MATLAB. The initial moment signal is the green phase, the simulation time step is 0.5 s, and the resolution of the vehicle trajectory profile is 0.1 s. The initial speed of the vehicle is 0 m/s, the initial acceleration is 0 m/s2, the desired speed through the intersection is 20 m/s, the vehicle spacing is 7 m, the initial headway distance is 3.5 s, and the desired headway distance is 1 s. The mass of the vehicle is 1200 kg, the coefficient of air resistance is 0.32, the air density is 1.185 kg/m3, the windward area of the vehicle is 2.5 m2, the coefficient of rolling resistance of the vehicle is 0.015, and the acceleration due to gravity is 9.8 m/s2: red light time 25s, green light time 25s. The simulation is conducted using MATLAB to verify the model’s effectiveness. The following are the parameters related to the simulation experiment (Table 3) [23].

**Table 3:** Parameters of simulation experiment.

CAV Penetration ρ	Expected Velocity $v_{e}/(m\cdot s^{-1})$	Maximum Velocity $v_{max}/(m\cdot s^{-1})$	Minimum Velocity $v_{min}/(m\cdot s^{-1})$	Maximum Acceleration $a_{max}/(m\cdot s^{-1})$	Minimum Acceleration $a_{min}/(m\cdot s^{-2})$${𝐚}_{min}$
20%	20	25	0	2	−2
40%	20	25	0	2	−2
60%	20	25	0	2	−2
80%	20	25	0	2	−2

## 4. Results and discussion

### 4.1 Simulation results and discussion of vehicle spatial and temporal trajectories

Compared with the spatio-temporal trajectories of mixed traffic flow generated by traditional signal control, those optimised by the two-stage asynchronous hierarchical optimisedion strategy proposed in this study (Fig 7). Under the two-stage asynchronous hierarchical optimisation strategy, connected and autonomous vehicles rely on on-board and roadside perception devices to obtain real-time traffic data via the cloud traffic information interaction system, and dynamically optimise vehicle motion parameters with the help of distributed information processing modules and vehicle-road cooperative communication network systems. This strategy ensures that vehicles can pass through intersections without stopping while maintaining relatively stable spatio-temporal trajectories, effectively reducing the number of stops and providing strong support for improving the traffic efficiency at intersections [23].

**Fig 7:**
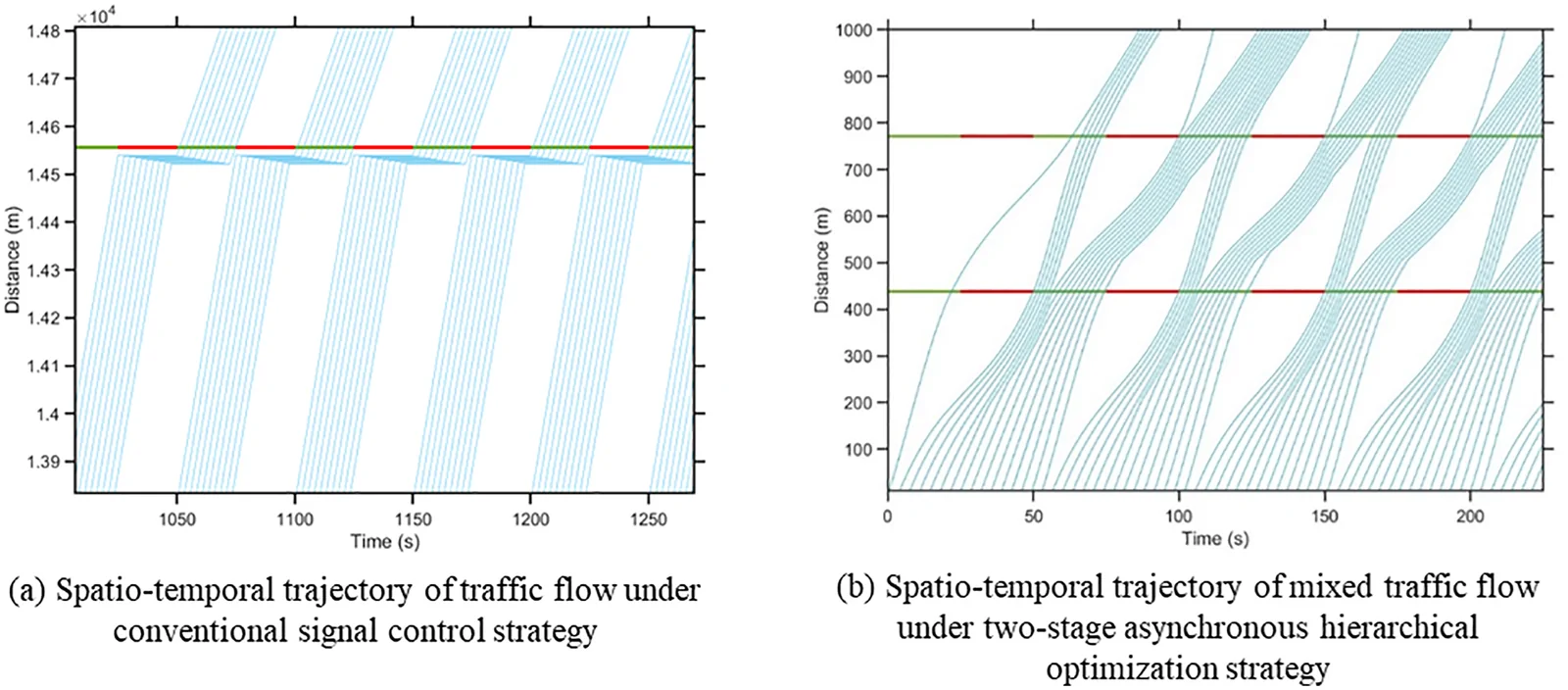
Spatial and temporal trajectory map of traffic flow simulation.

### 4.2 Vehicle speed simulation results and discussion

As the CAV penetration rate increases from 20% to 80%, the speed distribution of CCAV shows a clear central tendency, but the main peak speed increases as the CAV penetration rate rises from 20% to 60%. Under a high penetration rate, CCAVs can collaborate more efficiently, such as distance keeping, lane changing collaboration, etc., to reduce the blockage in the traffic flow, so that the CCAVs can travel at a more economical speed, improve the efficiency of road access, and shorten the travel time (Fig 8). The speed distribution of the CAV at each penetration rate is more dispersed and exhibits multi-peak characteristics, indicating that it can flexibly adjust its speed across different driving conditions. Still, due to the limitations of local optimisation, HDV is constrained by the inherent defects in human drivers’ decision-making and the diversity and uncertainty of human driving behaviours. Its speed distribution is most dispersed at each penetration rate, lacks an evident central trend, and shows more extreme values at both low and high speeds.

**Fig 8:**
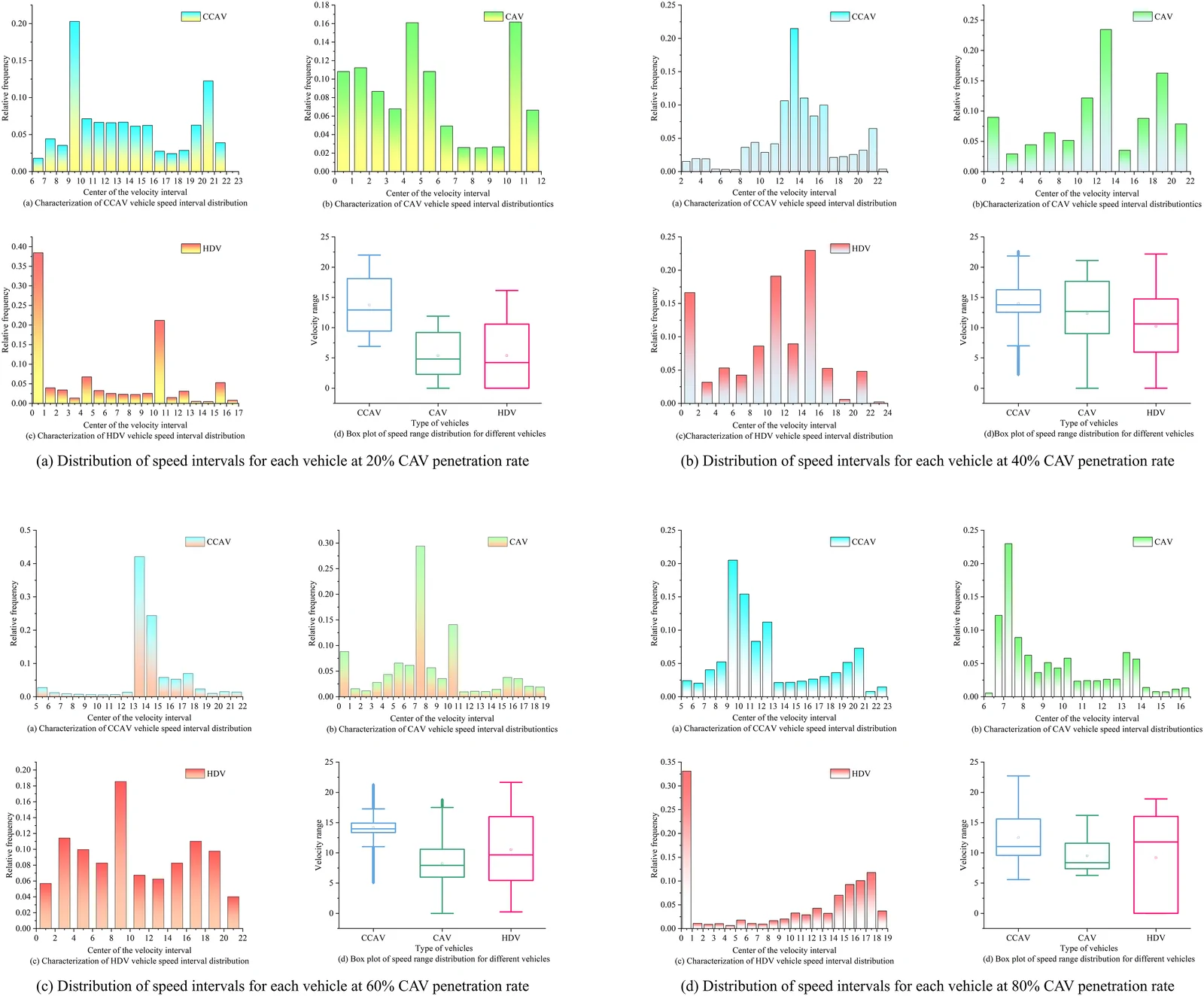
Vehicle speed interval distribution for different CAV penetration rates.

The average number of stops (when the speed is 0, considered as a stop) of the self-driving vehicle with the proposed trajectory optimisation strategy is lower than that of the human-driving vehicle in each scenario with different CAV penetration (Fig 9). When CAV penetration is 20%, HDV stops 4 times in mixed traffic flow, each lasting about 50–70 s; CAV stops 1 time, lasting about 40 s; and CCAV stops 0. When CAV penetration is 40%, HDV stops 3 times in mixed traffic flow, each lasting about 40 s; CAV stops 1 time, lasting about 35 s; and CCAV stops 0. When CAV penetration is 60%, the average number of stops is lower than that of human-driven vehicles. When CAV penetration is 60%, HDV stops 2 times in mixed traffic streams for about 15 seconds, CAV stops 1 time for about 20 seconds, and CCAV stops 0 times. When CAV penetration is 80%, HDV stops 2 times in mixed traffic streams for about 60–80 seconds, CAV stops 0, and CCAV stops 0. As CAV penetration increases, the number of stops for self-driving and human-driven vehicles decreases, indicating that the more CAVs there are in the road network, the greater the benefit to human-driven vehicles, and that self-driving vehicles can play a particular role in guiding HDV drivers. At the same time, the duration of stops is decreasing, and the percentage decrease continues to grow as CAV penetration increases, as shown in Table 4. The increase in CAV penetration can reduce traffic disturbances, increase HDV speeds, and optimise HDV following behaviour, allowing more vehicles to pass through the intersection in the same amount of time, thereby improving overall traffic performance at the intersection.

**Table 4:** Reduction in stops spaces.

	A 40% penetration rate for CAVs represents a decrease compared to 20%	A 60% penetration rate for CAVs represents a decrease compared to 20%	An 80% penetration rate for CAVs represents a decrease compared to 20%
Percentage reduction in CAV stops (%)	0	0	100
Percentage reduction in HDV stops (%)	25	50	50
Percentage reduction in CAV parking duration (%)	12.5	50	\
Percentage reduction in HDV parking duration (%)	>20	>70	Up 10

**Fig 9:**
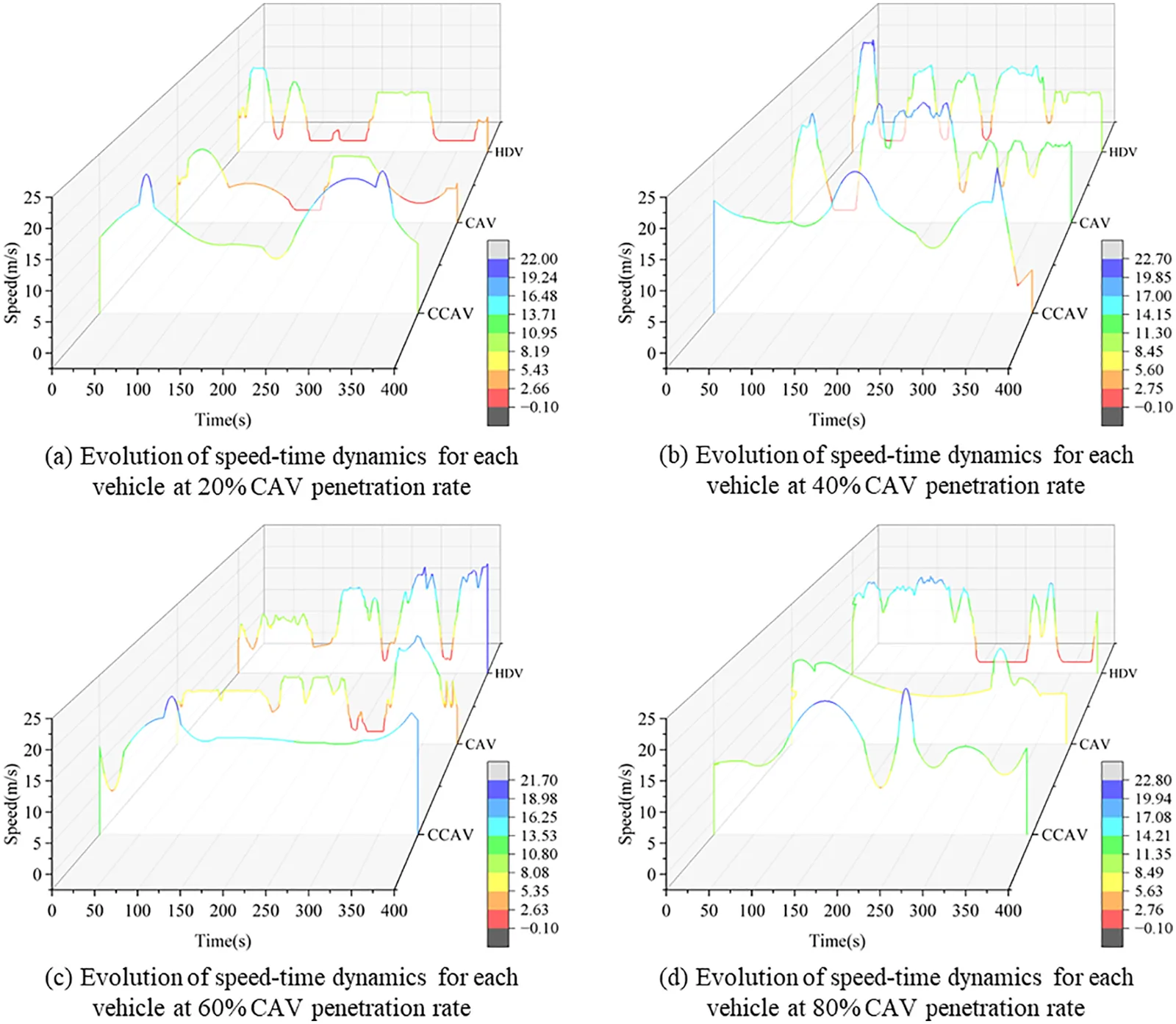
Evolution of speed-time dynamics for each vehicle with different CAV penetration rates.

Table 4 shows the improvement in parking metrics under different penetration rates of connected and automated vehicles (CAVs). Synergistic effects are particularly pronounced in high-penetration scenarios: when penetration reaches 80%, the number of CAV stops is reduced by 100%, effectively eliminating stopping behavior entirely. This is primarily due to smooth trajectory planning aided by vehicle-to-vehicle (V2V) communication. Conventional human-driven vehicles (HDVs) also benefit from the improved overall stability of the mixed traffic flow. At penetration rates of 60% and 80%, the reduction in HDV stopping incidents stabilizes at 50%, indicating that high-density intelligent vehicles can effectively dampen traffic waves and reduce the need for braking by human drivers behind them. Furthermore, regarding stopping duration, while there were brief increases in some scenarios (such as a 40% CAV penetration rate) due to adjustments in vehicle interaction strategies, at a 60% penetration rate, the stopping duration for CAVs and HDVs decreased significantly by 50% and over 70%, respectively. This further validates the effectiveness of this strategy in improving traffic efficiency and reducing unnecessary waiting time.

Table 5 shows that the average delay for CAVs and CCAVs at intersections exhibits a significant downward trend as CAV penetration increases: At a 20% penetration rate, delays remain at a relatively high level (approximately40–120 s/veh); when the penetration rate rises to 40% and 60%, delays decrease to approximately 20–90 s/veh and 20–40 s/veh, respectively; and under high-penetration conditions of 80%, delays further converge to around 20–30 s/veh; Although delays for HDVs increased, the overall increase was modest. This indicates that the proposed coordinated optimization strategy is particularly effective at improving intersection throughput under conditions of high CAV penetration.

**Table 5:** Changes in delays.

CAV Penetration ρ (%)	CCAV Delays (s)	CAV Delays (s)	HDV Delays (s)
20	49.1	126.4	2.2
40	28.8	88.3	7.7
60	38.1	24.7	6.6
80	8.3	5.8	14.7

### 4.3 Energy consumption simulation results and discussion

The energy consumption of self-driving vehicles with trajectory-optimisation strategies in mixed traffic is lower than that of manually driven vehicles across various scenarios with different penetration rates (Fig 10). The energy consumption of each vehicle type shows significant differences and exhibits a clear evolutionary trend as penetration rates increase.

**Fig 10:**
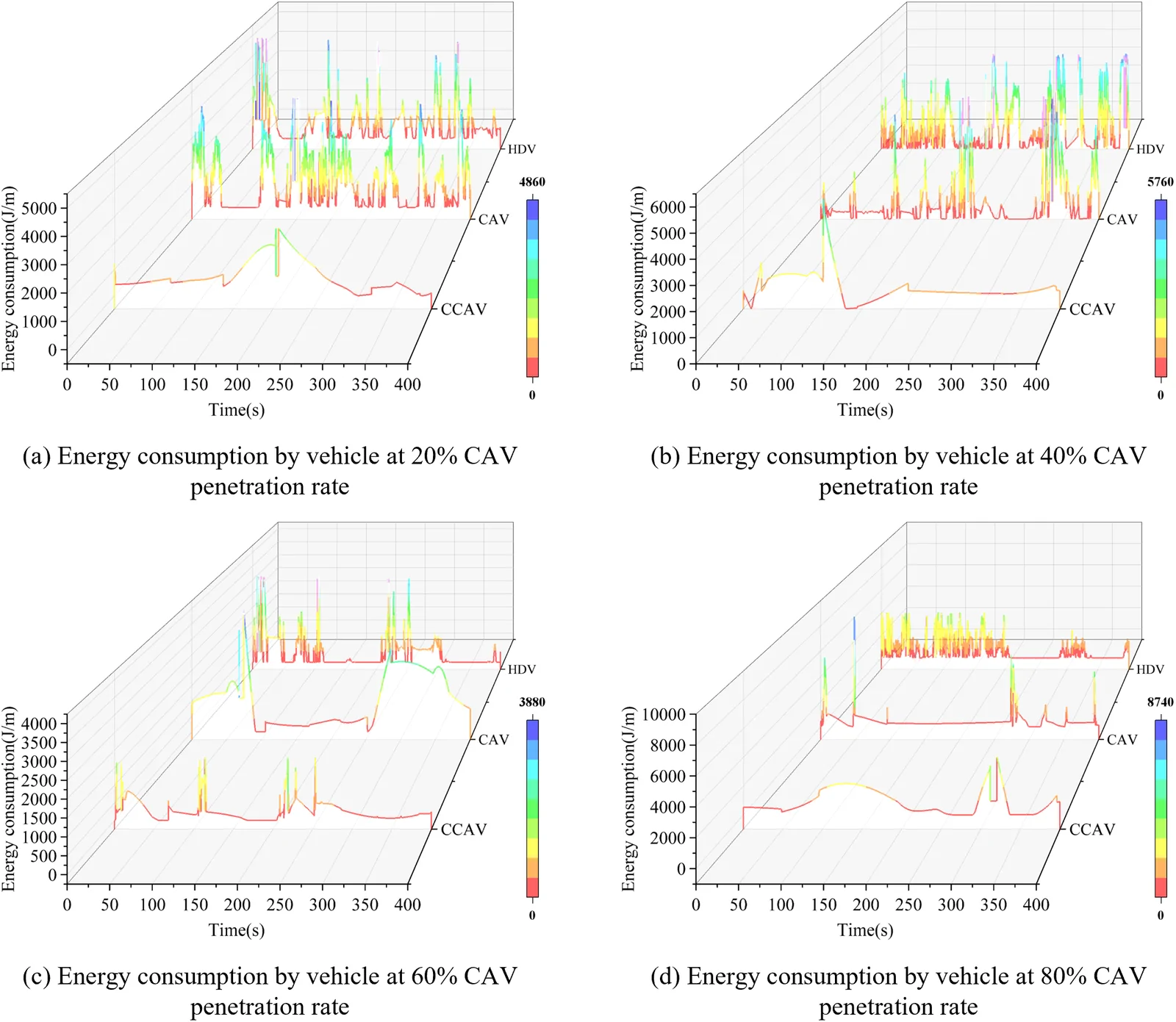
Energy consumption of each vehicle with different CAV penetration rates.

At 20% CAV penetration, CCAV energy consumption remains roughly within 0–3500 J/m, which is significantly lower than that of CAVs and HDVs.This difference mainly stems from the fact that CCAVs achieve speed smoothing through cooperative control strategies that reduce inefficient acceleration and braking. In contrast, HDVs are unable to integrate effectively into the cooperative flow, and their frequent sharp accelerations and braking result in higher energy consumption. As the CAV penetration rate increases to 40%, the maximum energy consumption of CCAV increases, the overall energy consumption of CAV decreases, the gap between the peak and the valley is reduced, the main energy consumption range is roughly in the range of 3000-5000J/m, and the energy consumption of HDV is also on an upward trend. This indicates that the initial formation of the cooperative network positively affects energy consumption optimisation. At the same time, whereas HDV frequently adjusts its speed to match the CAV group’s rhythm, leading to increased energy consumption. When the CAV penetration rate reaches 60%, the energy consumption of CCAV, CAV, and HDV further decreases, indicating that the multi-vehicle cooperative effect significantly improves the overall efficiency of traffic flow. At 80% CAV penetration, the energy consumption of CCAV and CAV is minimised, while that of HDV increases. It shows that deep optimisation of cooperative control algorithms at a high penetration rate significantly reduces the energy consumption of mixed traffic flow but exacerbates the energy degradation of HDVs to some extent. This is because CCAVs and CAVs share traffic-state information in real time via V2X communication, enabling globally optimal speed planning and trajectory optimisation.

Overall, CCAV energy consumption continues to decrease as CAV penetration increases, mainly due to the expansion of cooperative control and the increase in self-driving vehicles. The energy consumption of CAV gradually decreases after 40% penetration, with the inflexion point at 40%, indicating that the group synergy effect begins to emerge. In contrast, the energy consumption of HDVs decreases less with CAV penetration, mainly because HDVs themselves frequently adjust their speeds to adapt to CAV-dominated traffic flows, thereby increasing energy consumption. Overall, the increase in CAV penetration significantly improves the energy efficiency of cooperative vehicles, and the energy consumption of CCAV, CAV, and HDV decreases with increasing CAV penetration, thereby verifying the effectiveness of the two-phase asynchronous hierarchical optimisation strategy in reducing energy consumption.

At low penetration rates, the energy consumption of CAV and HDV fluctuates and is high; at high penetration rates, the fluctuations in all types of vehicles’ energy consumption decrease and the levels drop. However, at high CAV penetration rates, the energy consumption of HDVs does not minimise. Instead, it increases because cooperative vehicles can form a “green band”, i.e., significantly reduce ineffective acceleration and braking behaviours by coordinating their speeds. This synergistic effect leads to a continuous decrease in the energy consumption of CCAVs and CAVs as the penetration rate increases. However, due to a lack of synergy, HDVs cannot effectively integrate into CAV-dominated traffic flows, and human drivers often need to adjust their speeds frequently to match dynamic changes in traffic flow, given the stochastic and unpredictable nature of human driving behaviour. Such frequent speed adjustments lead to increased rapid acceleration and braking among HDVs, which, in turn, exacerbates energy waste.

As the penetration rate of connected autonomous vehicles increases from 20% to 80%, their speed-time trajectories show a clear trend toward smoothing. At a 20% penetration rate, manually driven vehicles still frequently exhibit sudden acceleration and deceleration, resulting in severe speed fluctuations and frequent stops—behaviors closely associated with high-energy-consumption driving patterns. However, when the penetration rate rises to 80%, connected autonomous vehicles effectively suppress speed fluctuations through cooperative adaptive cruise control, enabling continuous and smooth travel and significantly reducing unnecessary braking and re-acceleration. Based on the improved smoothness of the speed trajectories, we further calculated the average energy consumption per vehicle under different penetration rates. The results indicate that, compared to the 20% penetration rate scenario, average energy consumption decreased by approximately 32.6% at an 80% penetration rate. This significant reduction is primarily attributed to the coordinated control achieved by connected autonomous vehicles through vehicle-to-vehicle communication, which effectively avoids the “stop-and-go” phenomenon typical of traditional traffic flows, thereby substantially improving energy efficiency.

## 5. Conclusions

In this paper, the signalised intersection traffic problem in an intelligent network environment is studied, with the mixed traffic flow composed of CCAV, CAV, and HDV taken as the research object. The following model is established, and the two-stage cooperative optimisation algorithm is adopted to coordinate the driving states of each vehicle in the mixed fleet and to optimise the following distance and vehicle speed. The trajectory optimisation model integrates by combining traffic signals and vehicle energy consumption, thereby effectively reducing vehicle energy consumption. The strategy has good generalizability, and its effectiveness has been fully verified in signalised intersection scenarios. Its application scope is not limited to intersection scenarios; it can also be extended to other scenarios with similar traffic control mechanisms, enabling the strategy to leverage its optimisation advantages across many traffic scenarios and providing strong support for the application of intelligent transportation systems.

The main conclusions of this paper are as follows:

(1) The energy consumption of CCAV decreases continuously with the increase of CAV penetration, thanks to the expansion of the cooperative control range and the increase of self-driving vehicles. The energy consumption of CAV decreases gradually after 40% penetration, and the inflexion point occurs at 40%, indicating that the group synergy effect begins to emerge. On the other hand, the energy consumption of HDVs decreases less with increasing CAV penetration rate, which is exacerbated by their frequent speed adjustments when adapting to CAV-dominated traffic.
(2) The increase in CAV penetration significantly optimises the energy efficiency of cooperative vehicles. As the penetration rate increases, the energy consumption of CCAV, CAV decreases, verifying the effectiveness of the two-stage asynchronous hierarchical optimisation strategy in reducing energy consumption. The average delay for CAVs and CCAVs at intersections exhibits a significant down-ward trend as CAV penetration increases.
(3) At low penetration rates, the energy consumption of CAV and HDV fluctuates and has high values; at high penetration rates, the energy consumption of all types of vehicles fluctuates and decreases in level. However, at high CAV penetration rates, HDV energy consumption does not decrease; it increases instead. This is because the coordinated vehicles form a “green band”, which significantly reduces inefficient acceleration and braking by coordinating speeds, thereby enabling the energy consumption of CCAVs and CAVs to continue decreasing with increasing penetration.

Limitations and Future Work:

The primary objective of this paper is to propose and validate the “dynamic coupling mechanism in mixed traffic environments,” with a specific focus on the real-time interaction logic between vehicle trajectories and traffic signals under different CAV penetration rates, rather than conducting a comprehensive benchmarking of all possible control strategies. The selected baselines aim to establish a performance lower bound to demonstrate the theoretical feasibility of the proposed cooperative framework under ideal conditions. Regarding the use of multiple random seeds for statistical evaluation, this was a trade-off driven by computational resource constraints and the scope of the current submission. Given that our study employs high-fidelity micro-traffic simulation and a two-stage iterative optimization algorithm, the computational complexity of a single run is extremely high. To ensure that the algorithm’s complete convergence trend across the full penetration rate range (20%–100%) could be demonstrated within a feasible timeframe, we prioritized coverage of core scenarios. Although the current results demonstrate the deterministic nature of the representative runs, we have provided a detailed list of all model parameters and initial conditions to ensure reproducibility. We commit to incorporating experiments with multiple random seeds in future research and to providing detailed ablation baseline experiments and analyses.Overall, the eco-driving strategy can effectively reduce the number of Energy Consumption stops and delays at intersections, is generally applicable, can be extended to other scenarios with similar traffic control mechanisms, and provides strong support for the application of intelligent transportation systems.

## Supporting information

S1 File The “data_consumption” file contains simulated energy consumption data. (XLSX)

S2 File The file “data_t_v_p” contains simulated traffic data. (XLSX)

## Abbreviations

HDV: Human-Driven Vehicle
AV: Autonomous Vehicle
CAV: connected autonomous vehicles
CCAV: Cooperative Connected-Automated Vehicle
IDM: Intelligent Driver ModelACC, adaptive cruise control
CCAC: cooperative adaptive cruise control
